# Assessment of polytraumatized patients according to the Berlin Definition: Does the addition of physiological data really improve interobserver reliability?

**DOI:** 10.1371/journal.pone.0201818

**Published:** 2018-08-23

**Authors:** Carina Eva Maria Pothmann, Stephen Baumann, Kai Oliver Jensen, Ladislav Mica, Georg Osterhoff, Hans-Peter Simmen, Kai Sprengel

**Affiliations:** Department of Trauma, University Hospital Zurich, Zurich, Switzerland; Klinikum rechts der Isar der Technischen Universitat Munchen, GERMANY

## Abstract

**Background:**

Several new definitions for categorizing the severely injured as the Berlin Definition have been developed. Here, severely injured patients are selected by additive physiological parameters and by the general Abbreviated Injury Scale (AIS)-based assessment. However, all definitions should conform to an AIS severity coding applied by an expert. We examined the dependence of individual coding on defining injury severity in general and in identifying polytrauma according to several definitions. A precise definition of polytrauma is important for quality management.

**Methods:**

We investigated the interobserver reliability (IR) between several polytrauma definitions for identifying polytrauma using several cut-off levels (ISS ≥16, ≥18, ≥20, ≥25 points, and the Berlin Definition). One hundred and eighty-seven patients were included for analyzing IR of the polytrauma definitions. IR for polytrauma definitions was assessed by Cohen’s kappa.

**Results:**

IR for identifying polytrauma according to the relevant definitions showed moderate agreement (<0.60) in the ISS cutoff categories (ISS ≥16, ≥18, and ≥20 points), while ISS ≥25 points just reached substantial agreement (0.62) and the Berlin Definition demonstrated a correlation of 0.77 which is nearly perfect agreement (>0.80).

**Conclusion:**

Compared with the ISS-based definitions of polytrauma, the Berlin Definition proved less dependent on the individual rater. This underlines the need to redefine the selection of severely injured patients. Using the Berlin Definition for identifying polytrauma could improve the comparability of patient data across studies, in trauma center benchmarking, and in quality assurance.

## Introduction

Severe trauma is one of the tenth most common causes of death worldwide also in upper-middle income countries and killed 1.3 million people in 2015 in road accidents [1]. Among severely injured or “polytraumatized” patients, the impact on society is by far more than that in patients with isolated trauma. The polytrauma population needs to be accurately identified to allow appropriate reimbursement and benchmarking of trauma centers [2]. Coding represents the most important tool to compare the individual injury severity on defining the severity of injuries in general and in identifying a patient with polytrauma.

The Injury Severity Score (ISS) [3], based upon the Abbreviated Injury Scale (AIS) [4, 5], is the best-established score used for evaluating the severity of injury of multiply injured patients worldwide [3–6]. In practice, a major trauma is often defined by an ISS cutoff value of ≥16 points, but there are also other definitions using ISS cutoff values of ≥16 to ≥26 points [3, 7, 8]. Recently, Butcher and Balogh revealed that the underlying AIS coding alone may represent a basis to assess patient with multiple injuries. The authors describe that using the AIS as an anatomical basis to specify severely injured patients can identify patients at high risk for complications [9]. In literature for these cohort of patient’s numerous synonyms are used such as “critically ill patient with traumas”, “severely injured patients”, “patients with several injuries”, “multiply injured patients” or the term “polytrauma”. A recent definition of polytrauma is the Berlin Definition, which arose based on Butcher and Balogh’s assumptions, performed a consensus process and aimed to consider circumstances leading to high morbidity rates [9–12]. Moreover, it was found that a combination of the AIS and objective, quantitative, and measurable parameters was relevant in predicting mortality in patients with polytrauma [11–13].

Calculations of the AIS and ISS are based on comprehensive and complex coding rules. As this is usually performed by one person, it is subject to the individual’s evaluation of an injury, which might be incorrect and often varies between different institutions. Butcher et al. showed that the subjective definition of polytrauma can differ substantially within and between institutions [7].

Determining the optimal method to identify a patient as being polytraumatized is important, because trauma scoring has proved to be an important tool in the quality management of patients with acute injuries [14]. It is likely to become a model for “pay-for-performance” issues in the future. From this perspective, the interobserver reliability investigated in this study is an important tool for assuring good performance, even with economic factors in mind.

To ensure adequate comparability of the patient data across studies, for trauma center benchmarking and for quality assurance procedures, this human factor should be minimized.

The aim of the current study was to examine the dependence of individual injury severity coding on defining the severity of injury in general. Also, the advantage of the Berlin Definition in identifying a patient with polytrauma compared to the ISS-based grade, in respect to the rater dependence, was emphasized.

## Material and methods

### Study design

The basis for this study is a single-center database of polytrauma patients, which started in 1996 and was continued throughout. Moreover, our department has begun to participate in the documentation of the national trauma registry in 2009. Patients who were admitted via the resuscitation room with need for intensive care treatment were included. Every patient underwent a standardized whole-Body CT scan and cranial CT scan following a defined radiological protocol [15].

In 2014, the Berlin Definition of Polytrauma was published and the data base documentation of 2009 was reassessed between April 1, 2015 and December 01, 2015.

The regional institutional review board approved this study (Kantonale Ethikkommission Zurich, StV-01/2008, 20.11.2007). The need for consent from patients was waived because the database and the registry were anonymous. The study was conducted in agreement with the principles of the seventh revision of the Declaration of Helsinki including its clarifications and with Good Clinical Practice Guidelines.

### Independent documentation during a 1-year period

In the year 2009 two independent research groups performed all trauma coding, because in addition to coding for the internal polytrauma database (TDS group), a second group started coding independently for the national trauma registry (TR group). In 2010, both groups were merged, so the analysis could only be conducted with the data for this 1-year period.

The coding of the group TDS (internal polytrauma database) was performed by one doctoral student who coded every patient of the year 2009 under supervision of the senior author. In the group TR (national trauma registry) the dataset of 2009 was divided equally over four interns with at least 3 years of clinical experience (experienced physicians), every patients data was only coded once by one of the four interns under the supervision of the senior author.

The data sources for the calculation and coding were the patients’ electronic medical history (including all reports, documentation, findings reports as well as all x-rays, CT-scans and photos). The AIS calculation for every patient is based on the coded diagnoses in the discharge report, operation reports and transfer reports from the intensive care unit. In the case of unclear diagnoses, the x-rays and CT-scans were reviewed, and the radiological reports sighted. All research associates were trained in this technique by the senior author.

A specially trained doctoral student (TDS) coded all injuries under the supervision of the senior author. Coding includes the maximum injury severity according to the AIS [4, 5] of each body region (MAIS) for the head or neck, face, chest, abdominal or pelvic contents, extremities or pelvic girdle, or external injuries according the ISS regions and calculated autonomously the ISS [3], new ISS (NISS) [16], and the Trauma and Injury Severity Score (TRISS) [17] of the patients within the internal polytrauma database which was stored into a spreadsheet (Excel 2013, Microsoft Corp., Redmond, WA, USA).

The assessed element of TRISS in this study was the survival probability including age and mechanism of injury.

The trauma registry (TR) group (four interns with experience in polytrauma management and coding) coded the AIS of every injury in a web-based mask. Here, the calculations of MAIS, ISS, NISS, and TRISS scores were performed automatically by the registry. The registry data are provided annually in an IBM SPSS Statistics software (SPSS) database file.

Both the TDS and the TR group performed the coding under the supervision of the senior author (KS). The senior author attended a manufacturer`s course of the Association for the Advancement of Automotive Medicine (AAAM) to get trained in coding the AIS dictionary. The Abbreviated Injury Scale 2005 Update 2008 [4] was used for injury coding.

### Parameters of interest and definitions

The parameters used from the internal polytrauma database were age, gender, trauma pattern, body mass index, length of stay, length of stay in the intensive care unit, ventilator days, and mortality. The following parameters were recorded at admission: Glasgow Coma Scale (GCS) [18] (also using the first value recorded on the scene), temperature, heart rate, systolic arterial pressure (also using the first value recorded on the scene), hemoglobin level, hematocrit, partial thromboplastin time (PTT), international normalized ratio (INR), prothrombin time, base excess and lactate levels. The patient’s records were assessed to gain physiological data to calculate the TRISS and evaluate the Berlin Definition in addition to the assessed data of the dual injury codes.

Furthermore, the MAIS for different body regions [4], ISS [3], NISS [16], and TRISS [17] was extracted from the internal polytrauma database and from the trauma registry data set.

### Patient assessment based on existing data bases

Between 01.04.2015 and 01.12.2015, all patients from the 2009 patient sets were reassessed in a new SPSS database file. Identifying a patient as polytraumatized or not according to the ISS and the Berlin Definition was additionally coded automatically by absolute values or set parameters.

Therefore, three dichotomous variables in the TDS and TR group were generated according to the ISS only using a value of ≥16, ≥18,≥20 and ≥25 points. ISS cut off values of ≥16 and ≥25 points were chosen, because they are the most commonly used definition in literature and ISS ≥20 points, because in Switzerland it is the requirement to bring patients to one of the 12 authorized highly specialized medical polytrauma centers [1, 5, 6]. ISS ≥18 points was selected as an additional value between those two cut offs.

Another variable was created in the TDS and TR group identifying a patient as polytraumatized or not according to the Berlin Definition which consider not only the injury scoring, but also some physiologic parameters. The underlying scoring and parameters for identifying a polytraumatized patient are a MAIS value of ≥3 points of two body regions in combination with one physiological problem such as hypotension, defined as a systolic arterial pressure ≤90 mmHg on the scene or at admission, coagulopathy (PTT ≥40 seconds or INR ≥1.4), acidosis (base excess ≤–6.0 mmol/L), age ≥70 years, or unconsciousness with a GCS ≤8 points.

The exclusion criteria for analysis of polytrauma definitions in this study comprised missing paired injury coding, withdrawal of medical support within 24 hours and transfers from other hospitals. This was because the physiological parameters used for the Berlin Definition could otherwise be biased.

### Statistical analysis

Cohen’s kappa, with 95% confidence interval (95% CI), was used for identifying polytraumatized patients [19, 20]. Continuous variables are displayed as the median and range. Categorical data are summarized using numbers and percentages. The data were analyzed using IBM SPSS Statistics software (version 25.0.0.1; IBM Corp., Armonk, NY, USA). Graphs were created with GraphPad Prism (version 7.04; GraphPad Software Inc, La Jolla, CA, USA).

## Results

### Patient sample

**Fig 1:**
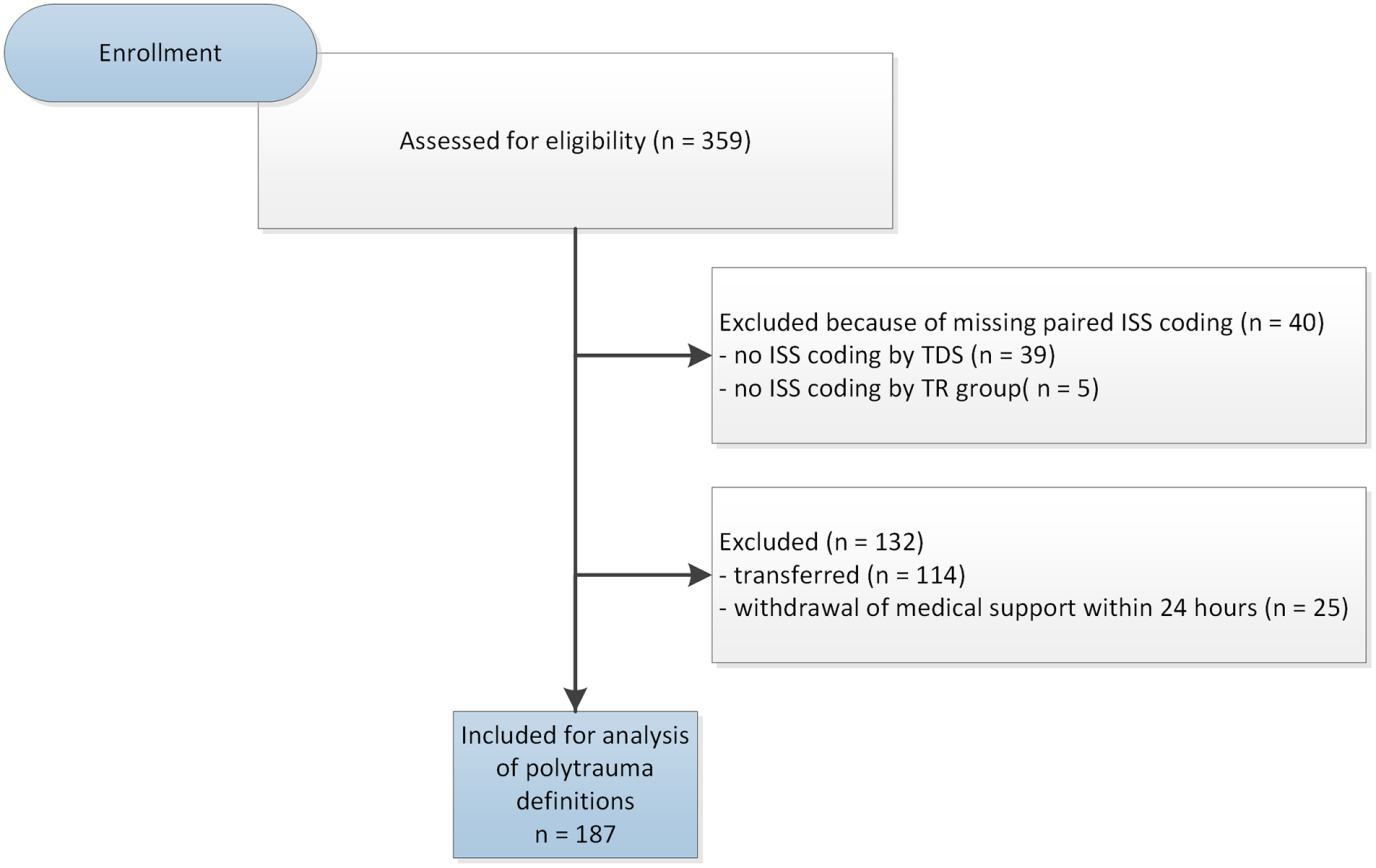
Selection procedure of the study population. ISS, Injury Severity Score; TDS, trained doctoral student; TR, trauma registry.

A total of 359 patients were considered for the study, 187 patients were used for analysis of the definitions of polytrauma. The selection procedure is shown in Fig 1, and an overview of the sample population is given in Tables 1 and 2. A glossary of all used abbreviation is available in S1 Table.

**Table 1:** Baseline data used for the analysis of interobserver reliability.

Parameter	Polytrauma definitions
Number of patients	187
Age (years)	45.0 (16–90)
Gender male/female: *n* (% male)	134/53 (71.7)
ISS (points)	25 (4–75)
NISS (points)	30 (4–75)
ISS ≥16 and 2 regions MAIS ≥2: *n* (%)	131 (70.1)
MAIS_head and neck_ ≥5: *n* (%)	56 (29.9)
BMI (kg/m^2^)	24.6 (17.1–47.0)
Blunt trauma mechanism: *n* (%)	165 (88.2)
Primary admission/transferred: *n* (%)	187 (100) / 0 (0)
Hospitalization (days)	11.0 (1–97)
ICU stay (days)	2.0 (0–65)
Ventilator (days)	1.0 (0–33)
Mortality rate: *n* (%)	34 (18.2)
At admission	
GCS (points)	14 (3–15)
Temperature (°C)	35.8 (31.7–38.7)
Heart rate (bpm)	85.0 (50.0–160.0)
SAP (mmHg)	135.0 (45–205)
Hemoglobin (g/dL)	13.0 (3.4–16.9)
Hematocrit (%)	39.0 (13.6–51.7)
INR	1.1 (0.9–3.2)
Prothrombin Time (%)	87.0 (20–100)
Base excess (mmol/L)	-2.6 (-18–5.5)
Lactate (mmol/L)	2.1 (0.4–9.1)

ISS, Injury Severity Score; NISS, New ISS; MAIS, maximum Abbreviated Injury Scale; GCS, Glasgow Coma Scale; ICU, Intensive Care Unit; BMI, body mass index; SAP, systolic arterial pressure; bpm, beats per minute; INR, international normalized ratio. ISS, NISS and AIS based on the trained doctoral student coding. Continuous variables are displayed as medians and (range), categorical data as numbers and (percentages).

**Table 2:** Baseline data depending on AIS Coding.

Parameter	TDS group	TR group
Number of patients		
Total population	319	319
Polytrauma definitions	187	187
ISS (points)	25 (4–75) 25 (4–75)	22 (4–75) 21 (4–57)
NISS (points)	33 (4–75) 30 (4–75)	27 (4–75) 25 (4–66)
TRISS	0.970 (0.006–0.996) 0.966 (0.181–0.996)	0.956 (0.029–1.000) 0.962 (0.029–1.000)
ISS ≥16 and 2 regions MAIS ≥2: *n* (%)	219 (68.7) 131 (70.1)	172 (53.9) 95 (50.8)
MAIS_head and neck_ ≥5: *n* (%)	92 (28.8) 56 (29.9)	76 (23.8) 39 (20.9)
Polytrauma ISS ≥16: *n* (%)	273 (85.6) 160 (85.6)	237 (74.3) 132 (70.6)
Polytrauma ISS ≥18: *n* (%)	226 (70.8) 134 (71.7)	190 (59.6) 112 (59.9)
Polytrauma ISS ≥20: *n* (%)	216 (67.7) 125 (66.8)	174 (54.5) 100 (53.5)
Polytrauma ISS ≥25: *n* (%)	167 (52.4) 97 (51.9)	130 (40.8) 73 (39.0)
Polytrauma Berlin Definition: *n* (%)	77 (24.1) 49 (26.2)	72 (22.6) 45 (24.1)

TDS, trained doctoral student; TR, trauma registry; ISS, Injury Severity Score; NISS, New ISS; MAIS, maximum Abbreviated Injury Scale. Continuous variables are displayed as medians and (range), categorical data as numbers and (percentages).

### Interobserver reliability of the polytrauma definitions

The interobserver reliability of identifying a patient as having polytrauma according to the different definitions showed moderate agreement between raters with Cohen’s kappa coefficients of 0.41–0.60 as the reference when using the ISS: ISS ≥16 (0.425), ISS ≥18 (0.579), ISS ≥20 (0.573), and ISS ≥25 points (0.618). When using the Berlin Definition, there was substantial close to almost perfect agreement (0.773; almost perfect >0.80).

When comparing to the entire study population of 319 patients the results are nearly identical. ISS ≥16 (0.521), ISS ≥18 (0.537), ISS ≥20 (0.534), ISS ≥25 points (0.571) and Berlin Definition (0.781).

**Fig 2:**
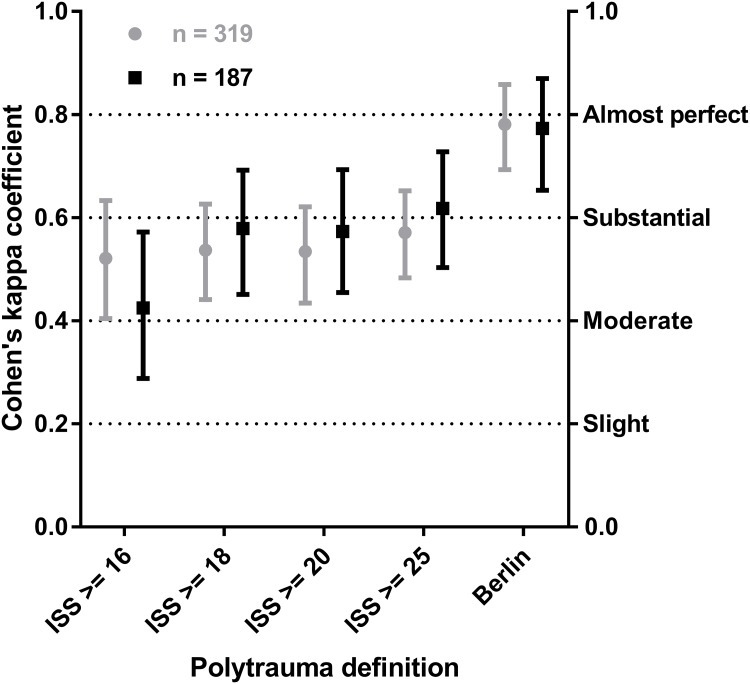
Interobserver reliability of the polytrauma definitions. Cohen’s kappa correlation coefficient with 95% confidence interval (95% CI); ISS, Injury Severity Score.

The results of the analysis of the interobserver reliability of the definitions of polytrauma are listed in Tables 3 and 4 and Fig 2.

**Table 3:** Interobserver reliability of the polytrauma definitions.

Definition	Number	Kappa	95% CI
ISS ≥16	187 (100%)	0.425	0.288–0.572
ISS ≥18	187 (100%)	0.579	0.451–0.692
ISS ≥20	187 (100%)	0.573	0.455–0.693
ISS ≥25	187 (100%)	0.618	0.503–0.728
Berlin Definition: two body regions with MAIS ≥3 plus one physiological problem	187 (100%)	0.773	0.653–0.870

Kappa, Cohen’s kappa coefficient; 95% CI, 95% confidence interval; ISS, Injury Severity Score; MAIS, Maximum Abbreviated Injury Scale.

**Table 4:** Degree of strength according to the interobserver reliability.

Kappa	Degree of strength^16^
<0.00	Poor
0.00–0.20	Slight
0.21–0.40	Fair
0.41–0.60	Moderate
0.61–0.80	Substantial
0.81–1.00	Almost perfect

Kappa, Cohen’s kappa coefficient.

## Discussion

Our cohort reflects a typical population of patients with blunt trauma like it is typical in Europe, among whom 61% were male patients, with a median age of 46 years. With the median ISS being 25 points and 70% of all patients having an ISS ≥16 points and also two body regions with a MAIS ≥2 points corresponding to 30% with a severe traumatic brain injury which could be responsible for a high ISS alone, a severely injured cohort is representative of those named “polytraumatized” as pointed out by Paffrath et al. [12].

Only moderate agreement between coders was found when using different ISS values as cutoff limits to identify a polytraumatized patient. The degree of agreement was lowest at ISS ≥16 points. For the definitions ISS ≥18, ISS ≥20, and ISS ≥25 points there were no obvious differences in agreement between coders but a tendency to better agreement in ISS ≥25 points. In contrast, the Berlin Definition showed substantial close to almost perfect agreement. For the ISS, this finding is consistent with Butcher et al., who reported that trauma surgeons do not agree on the definition of polytrauma, as they found only fair-to-moderate agreement on the subjective definition within and across institutions with kappa scores of 0.50 and 0.41, respectively [7]. This lack of agreement is also consistent with an investigation of the routine coding used in the Queensland Trauma Registry by Neale et al., who reported an intraclass correlation coefficient (ICC) for the ISS of 0.90, despite a relatively low level of agreement between coders for the AIS [21]. Several other studies found that the injury coding using the AIS was subject to variation between observers [8, 22, 23]. One reason could be that the additional physiological parameters included in the Berlin Definition reduce the differences between different coders and provide better comparability, because these physiological parameters are clearly defined and allow a clear allocation. Another reason might be that the calculation of the ISS can be difficult. As an example of the discussion concerning the ideal cutoff if using the ISS to identify a patient with polytrauma, a recent registry study of nearly 400,000 patients showed that for children compared with adults, the optimal cutoff was ISS ≥25 points [24]. We cannot address this issue here, because there were few children included in our study, but it emphasizes the point that there is an ongoing need for discussion of the ISS ≥16 points cutoff value for defining polytrauma and of all ISS-based classification the interobserver reliability was best in ISS ≥25 points.

One advantage of the Berlin Definition is that it uses the MAIS definitions, which in our study showed better agreement between experts than the ISS, which is consistent with Brown et al. [24]. Consequently, ISS-based definitions might give better results by automatic estimation of the ISS, and perhaps to some extent the MAIS for the Berlin Definition, as is done in some registries. This argument will be supported by Waydhas et al., who described significant deviation of the score data acquisition between raters of different professions or levels of education [10]. Therefore, despite automatic estimation, the benefit of training in injury coding using the AIS to reduce interobserver variability has been stressed by them [25].

The ISS is one of the most common systems used for evaluating polytrauma. It has been reported that the probability of any two raters associating the same ISS as 28%–51% [22]. Another study of 10 raters showed that the limit of agreement for each rater’s pairing crossed the “clinically acceptable” boundary, and that interobserver agreement for specific assigned ISS codes might be as low as 39% [21, 26]. Thus, compared with the ISS-based definitions of polytrauma, the Berlin Definition is less dependent on the person doing the rating.

### Limitations and strengths

This study had several limitations. First, it had a retrospective design and was not initially set up as a two-armed interobserver reliability study. It takes advantage of the coincidence, that in 2009 two separate groups performed the coding. Important preconditions, such as an equal level of training of the coders, could not be met. Second, it was a database investigation and the data were not collected specifically for this study.

We did not examine the precision of the rating personnel while coding, and none of the investigators had been certified in AIS methodology. However, their work was supervised and reviewed by the senior author, who is a licensed AIS coder. The interobserver reliability within the different coding personnel was not assessed. Furthermore, we did not identify potential bias that arose from the different professional backgrounds of coding physicians and the doctoral student. However, in contrast to Waydhas et al., Zoltie and De Dombal found no significant difference in the coding performance between experienced and inexperienced raters, and so the importance of this issue remains unclear [10, 22].

Our study also had important strengths. Among them was the prospective documentation of all data, the independent documentation by a group of experienced physicians, and the ability to control the data from the same hospital. Moreover, there was internal and external quality control, as determined by the registry and the senior author, who reviewed all cases personally.

## Conclusions

Compared with the ISS-based polytrauma definitions, the Berlin Definition is less reliant on the individual rater.

Our investigation underlines the current discussion on the need for a redefinition of the term “polytrauma” with respect to rater independence. If possible, the MAIS and ISS codes should be estimated automatically. We consider that the purely ISS-based polytrauma definitions, such as the ISS ≥16 value, should be abandoned in favor of the Berlin Definition, as this could guarantee better comparability of patient data across studies, in trauma center benchmarking, and in quality assurance procedures.

## Supporting information

S1 Table Glossary of abbreviations. (PDF)
