# Supplementary material for: Assessment of polytraumatized patients according to the Berlin Definition: Does the addition of physiological data really improve interobserver reliability?
Source: PLoS One. 2018 Aug 23;13(8):e0201818. doi: 10.1371/journal.pone.0201818 (PMC6107114; doi:10.1371/journal.pone.0201818)
Supplement: S1 Table — (PDF) [file pone.0201818.s001.pdf]

S1 Table. Glossary of abbreviations

AAAM	American Association for Automotive Medicine
AIS	Abbreviated Injury Scale
bpm	beats per minute
BMI	body mass index
GCS	Glasgow Coma Scale
ICC	intraclass correlation coefficient Cronbach's alpha
ICU	Intensive Care Unit
INR	international normalized ratio
IR	interobserver reliability
ISS	Injury Severity Score
Kappa	Cohen's kappa coefficient
MAIS	maximum injury severity related to the AIS of each body region according to the ISS regions
NISS	New Injury Severity Score
PTT	partial thromboplastin time
TDS	trained doctoral student
TR	trauma registry
TRISS	Trauma and Injury Severity Score
SAP	systolic arterial pressure
